# Perforated Appendicitis: An Unintended Consequence During the Coronavirus-19 Pandemic

**DOI:** 10.1093/milmed/usaa527

**Published:** 2020-12-04

**Authors:** Andrew W Wang, James Prieto, Daniel S Ikeda, Paul R Lewis, Emily M Benzer, Jan-Michael Van Gent

**Affiliations:** Departmet of Surgery, United States Naval Hospital Okinawa, Okinawa, Japan, 96362; Department of Surgery, Naval Medical Center San Diego, San Diego, CA 92134, USA; Departmet of Surgery, United States Naval Hospital Okinawa, Okinawa, Japan, 96362; Departmet of Surgery, United States Naval Hospital Okinawa, Okinawa, Japan, 96362; Departmet of Surgery, United States Naval Hospital Okinawa, Okinawa, Japan, 96362; Departmet of Surgery, United States Naval Hospital Okinawa, Okinawa, Japan, 96362

## Abstract

**Background:**

The coronavirus disease 2019 (COVID-19) pandemic has had major clinical impact across the globe. Delayed presentation for medical emergencies has been noted by the medical community. There has been limited reporting on the impact for the care for emergent surgical conditions. We sought to describe the effect of the global pandemic on the presentation and outcomes for the most common urgent general surgery disease process, acute appendicitis.

**Methods:**

We performed a retrospective review of patients admitted to the United States Naval Hospital Okinawa during the COVID-19 pandemic, from January 2020 to May 2020 (COVID cohort), and compared them to a historical cohort (pre-COVID cohort) over the prior 2 years. Demographics, clinical presentation data, and interventions were collected.

**Results:**

Of the 80 patients with appendicitis, 20% presented perforated. Most patients were male (71%), presented with 1 day of symptoms and had a length of stay of 1 to 2 days. Comparing groups, 13% of the pre-COVID group vs. 31% of the COVID cohort presented perforated (*P* = .04), with a symptom duration of 1.6 vs. 2.7 days before presentation (*P* = .075), respectively.

**Conclusions:**

The COVID-19 pandemic and the global systematic response has impacted unrelated medical and surgical conditions. At our overseas military hospital with minimal disease burden, we observed a delay in presentation for acute appendicitis with a higher incidence of perforation. Patients should be empowered to continue to seek care for urgent and emergent medical and surgical conditions so that they are not harmed by fear of COVID-19 rather than by COVID-19 itself.

## INTRODUCTION

The clinical impact of coronavirus disease 2019 (COVID-19) has been felt in healthcare systems across the globe as hospitals have shifted resources in an effort to meet the influx of critically ill patients. In January 2020, the first known case of COVID-19 was documented in the United States.^1^ Shortly thereafter, following guidance from the Surgeon General and the Center for Disease Control and Prevention, health systems, hospitals, and surgeons were encouraged to consider the cancellation or postponement of elective procedures in an effort to preserve both personal protective equipment and hospital infrastructure for the care of those ill with COVID-19.^2^ To date, there have been 4 million cases of COVID-19 in the United States and over 140,000 deaths attributed to the disease.^3^ However, while regional hotspots have required a massive influx of healthcare utilization dedicated to a COVID-19 response, there continues to be a need to deliver care for non-COVID-19-related medical and surgical emergencies. Many physicians have reported the “collateral damage” of unrelated conditions, where typical standards of care have been subverted in response to the current pandemic.^4,5^

The threat of emergency department COVID-19 exposure has not changed the case volume of percutaneous coronary intervention in the emergent care of myocardial infarction, for example, but has changed the symptom-to-door time as patients delay seeking care.^6^ In the care of cardiac emergencies, routine pathology can evolve into more complicated disease as the natural history of disease is allowed to progress at home for fear of COVID-19.^7^ Presumably, delays in presentation and/or care for other time-sensitive disease processes, including acute appendicitis, are also occurring. While it is generally accepted that the risk of tissue ischemia, gangrene, and perforation rises within 48-72 hours after symptom onset, there has been minimal reporting on the impact of the COVID-19 pandemic on rates of perforated appendicitis subsequent to delayed presentation for care. Perforated appendicitis leads to increased surgical morbidity, infectious complications, duration of hospitalization, and cost.^8^

The United States Naval Hospital Okinawa (USNHO) is the largest overseas military treatment facility that provides care for approximately 50,000 personnel on the island of Okinawa and nearly 200,000 patients and Tricare beneficiaries in the Western Pacific. Over 500 general surgery procedures are performed at the facility annually including over 100 appendectomies. As of May, the prefecture of Okinawa recorded 143 confirmed cases of patients with COVID-19 within the indigenous population and 4 cases in the military beneficiary population. Despite a negligible local disease burden, social distancing and telehealth were utilized to limit nonessential patient encounters and mitigate disease transmission within the facility. These well-intended interventions have undoubtedly made an impact on patients’ decision-making when it comes to seeking urgent medical evaluation. We hypothesized that during the COVID-19 pandemic, there was a delay in presentation and an increased incidence of perforation in patients presenting with appendicitis at our institution. The purpose of this study was to analyze the incidence of perforated appendicitis during the COVID-19 pandemic in order to better prepare for the delivery of urgent and emergent care for non-COVID-19 conditions during a potential second wave of COVID-19.

## METHODS

After Institutional Review Board approval was obtained (NMCSD.QI.2020.061101), we performed a retrospective review of all patients admitted to the USNHO with acute appendicitis between specified time periods related to the first wave of COVID-19 in the United States, as much of the policy for the military response to the COVID-19 pandemic for the island of Okinawa was guided by decisions made in the CONUS. This “COVID-19 cohort” was assigned to admissions beginning January 21, 2020, the date of the first documented COVID-19 case in the United States, until May 6, 2020. This was compared to our “historical cohort” of admissions between January 21, 2018 to May 6, 2018, and January 21, 2019 to May 6, 2019, the same time period the previous 2 years. The decision to compare the same time period in the previous calendar years was made due to predictable fluctuation in clinical volume throughout the calendar year in the military community as service members and their families move between duty stations.

The United States Naval Hospital Okinawa does not have interventional radiology capability, and all patients underwent appendectomy within 8 hours of admission unless the decision was made to pursue nonoperative management based upon clinical judgment. The diagnosis of perforated appendicitis was determined based on operative report and/or CT scan for patients managed nonoperatively. Demographic and perioperative data were collected and analyzed. Variables examined included age, sex, admission white blood cell (WBC) count, length of stay, and conversion to open procedure (*n*, %). The primary outcome for the study was the rate of perforated appendicitis (*n*, %), and the secondary outcome was the symptom duration before admission (days).

Statistical analysis was performed using IBM SPSS Statistics 25. Patient cohorts were compared using chi-square test, Student’s *t*-test, and Mann–Whitney U test where appropriate. *P*-values <.05 were considered significant.

## RESULTS

The COVID-19 cohort included 32 cases of appendicitis who received care at the USNHO. The historical cohort consisted of 48 cases of appendicitis at our institution. Overall, the mean age of the entire population was 26.3 ± 9.5 years, and the gender was more frequently male (71%). Median symptom duration was 1 day before presentation with a median length of stay of 1 day (Table I). The mean WBC count on admission was 12,600/L. Sixteen patients (20%) were diagnosed with perforated appendicitis.

**TABLE I.** Sample Characteristics of All USNHO Appendicitis Patients

Sample characteristics	
*n*	80
Age (years), mean (SD)	26.3 (9.5)
Female gender, *n* (%)	23 (29)
Symptom duration (days), median (interquartile range)	1 (1-2)
Symptom duration (days), mean (SD)	2 (2.7)
Perforated appendicitis, *n* (%)	16 (20)
WBC on admission, mean (SD)	12.6 (4.5)
Length of stay (days), median (IQR)	1 (1-2)
Conversion to open procedure, *n* (%)	2 (2.5)

Abbreviation: USNHO, United States Naval Hospital Okinawa.

In comparison with a historic cohort, there were no significant differences in age, gender, median symptom duration, admission WBC count, length of stay, or need for conversion to open procedure (Table II). However, 31% of cases of appendicitis in the COVID-19 cohort were perforated compared to 13% in the historical cohort (*P* = .04). There was a greater than 1 day delay in presentation after symptom onset in the COVID-19 cohort compared to the historical cohort, although this was not statistically significant (2.7 vs. 1.6 days, *P* = .075).

**TABLE II.** Comparison Between Historic Cohort and COVID-19 Cohort

	Historic cohort (*n* = 48)	COVID-19 cohort (*n* = 32)	*P*-value
Age (years), mean (SD)	27.3 (10.5)	24.8 (7.7)	.205
Female gender, *n* (%)	13 (27)	10 (31)	.687
Symptom duration (days), median (interquartile range)	1 (1-2)	1 (1-2.8)	.818
Symptom duration (days), mean (SD)	1.6 (0.9)	2.7 (4.1)	.075
Perforated appendicitis, *n* (%)	6 (13)	10 (31)	.04
WBC on admission, median (IQR)	12.2 (10.2-14.8)	12.4 (9.8-15.5)	.928
Length of stay (days), median (IQR)	1 (1-2)	1 (1-2.8)	.631
Conversion to open procedure, *n* (%)	1 (2)	1 (3)	.77

Abbreviation: WBC, white blood cell.

## DISCUSSION

There is no doubt that COVID-19 is a global pandemic unlike any seen before in recent history. In many places across the globe, including New York City, the virus has made many at-risk patients critically ill and in some cases has proven lethal.^9^ The response to such a threat requires an aggressive, carefully planned, and measured response by healthcare systems in order to allocate limited resources (personal protective equipment, ventilators, intensive care unit beds, etc.) and manpower to deliver exceptional and safe care to as many patients as possible. Additionally, public health authorities must make important and difficult decisions at a population level in an effort to “flatten the curve” to protect healthcare systems from being overwhelmed.^10^ These measures include use of telehealth resources, cancellation or postponement of elective procedures, and limits on access to care for conditions deemed nonurgent.^11^

The United States Naval Hospital Okinawa is the largest overseas hospital in the U.S. Navy providing primary and limited specialty care for the DoD personnel in the Western Pacific. Our facility is a freestanding hospital with 86 beds including 8 intensive care beds, a 2-bed trauma bay, 10 operating rooms, and a surge capacity of 130 beds. The facility approximates the capability of a level II trauma center within the CONUS. During the “first wave” of COVID-19 on the island of Okinawa, the USNHO implemented protocols to minimize or prevent the potential spread of disease within the facility including implementation of virtual patient encounters, telework for nonessential personnel, and establishment of a respiratory clinic, among other measures. Elective surgical procedures were canceled, and the access to surgical care was limited significantly in an effort to preserve personal protective equipment for the care of potential COVID-19 patients and for the care of urgent surgical and trauma patients. These efforts to minimize unnecessary traffic through the healthcare facility and limit interactions between those sick with respiratory illness and the general patient population resulted in a significant (nearly 50%) reduction in emergency department patient encounters, for example, as patients sought to follow hospital recommendations.

During this period, the department of general surgery experienced a clinically and statistically significant increase in the number of patients with perforated appendicitis at the time of presentation. Approximately one-third of the cases of appendicitis were perforated at the time of presentation compared to just over one-tenth during the same period of time in prior years. Additionally, while the average time to presentation did not achieve statistical significance likely because of the small sample size and insufficient power to detect such a difference, there was a trend toward significance. Anecdotally, during this period, it was not uncommon for patients to report they had considered seeking care earlier, but were trying to avoid the hospital because of fear of COVID-19 exposure. Fortunately, there was a similar duration of hospitalization and need for conversion to an open procedure between the COVID-19 cohort and the historical cohort despite this increased incidence of perforated disease. However, the fact remains that perforated appendicitis is associated with greater resource utilization and surgical morbidity.^8^

Our results are similar to other reports examining the impact of the COVID-19 pandemic of the presentation for evaluation for appendicitis. For example, a radiology group in Columbia found a decrease, during the COVID-19 pandemic, in the utilization of axial imaging to confirm the diagnosis of appendicitis along with a concordant increase in both the rate of positive CT scans and in the radiographic severity of disease detected.^12^ This suggested that fewer patients are presenting early for evaluation for appendicitis resulting in fewer early scans and more severe disease at presentation. Our findings also support those reported by Velayos et al.,^13^ which demonstrated an increase in the incidence of perforated appendicitis during the pandemic. In a pediatric cohort from Israel with complicated appendicitis related to delayed presentation, fear among patients and their families of exposure to COVID-19 in the healthcare environment was cited as the presumed reason for delay.^14^ All of these investigations come from areas around the world in which there is measurable community spread of COVID-19. Our data come from a time and environment in which the community spread on the island of Okinawa was minimal and there were zero confirmed cases of COVID-19 requiring hospitalization at our facility. A prolonged societal posture of “lockdowns” and care access limitations could result in real harm for patients requiring care for non-COVID-19 emergencies simply from the threat of disease even in the absence of confirmed disease.

There is no doubt that there must be a carefully planned and measured response among healthcare providers, medical systems, and public health departments in response to the COVID-19 pandemic. Patients who seek emergency medical care in place of a primary care provider for conditions that are unlikely to require an intervention should absolutely be encouraged to stay home. Additionally, there has been investigation into the role of antibiotics alone in the treatment of appendicitis as a temporizing measure to allow for definitive care following the resolution of the global pandemic.^15–18^ However, the threat of COVID-19 should not interfere with the delivery of safe and effective care for non-COVID-19 conditions. It is equally important that patients with urgent surgical conditions be encouraged to seek early and prompt medical care. While COVID-19 may have atypical presentations or even masquerade as appendicitis, it is vitally important to educate patients that life-threatening conditions such as myocardial infarction, stroke, trauma, and even appendicitis continue to occur amid a global pandemic.^19–22^ Patients must be empowered to continue to seek care for urgent and emergent conditions so that they are not harmed from fear of COVID-19 rather than by COVID-19 itself. As we prepare for a second wave of COVID-19 or any other respiratory illness of pandemic proportions, the delivery of safe, reliable, and timely care for other noninfectious, yet life-threatening emergencies remains of paramount importance.

## CONCLUSIONS

During the COVID-19 global pandemic, fear of exposure to the viral illness can impact the timing of presentation and resultant disease severity in the care of non-COVID-19 conditions. For appendicitis, delayed presentation can lead to higher rates of perforation resulting in increased morbidity. Patients must be empowered to continue to seek timely care for noninfectious emergent conditions despite a global pandemic.

## References

[1] Holshue ML, DeBolt C, Lindquist S, et al: First case of 2019 novel coronavirus in the United States. N Engl J Med 2020; 382(10): 929-36. 32004427 10.1056/NEJMoa2001191 PMC7092802

[2] COVID-19: recommendations for management of elective surgical procedures. Available at https://www.facs.org/covid-19/clinical-guidance/elective-surgery; accessed July 22, 2020.

[3] Dong E, Du H, Gardner L: An interactive web-based dashboard to track COVID-19 in real time. Lancet Infect Dis 2020 5; 20(5): 533-4. doi: 10.1016/S1473-3099(20)30120-1. 32087114 PMC7159018

[4] Gilligan J, Gologorsky Y: Collateral damage during the coronavirus disease 2019 (COVID-19) pandemic. World Neurosurg 2020 8; 140: 413-4. doi: 10.1016/j.wneu.2020.05.091. 32419877 PMC7224664

[5] Rosenbaum L: The untold toll — the pandemic’s effects on patients without COVID-19. N Engl J Med 2020; 382(24): 2368-71. 32302076 10.1056/NEJMms2009984

[6] Toner L, Koshy AN, Hamilton GW, Clark D, Farouque O, Yudi MB: Acute coronary syndromes undergoing percutaneous coronary intervention in the COVID-19 era: comparable case volumes but delayed symptom onset to hospital presentation. Eur Heart J Qual Care Clin Outcomes. 2020 Jul 1; 225-6. doi: 10.1093/ehjqcco/qcaa038. 32379888 PMC7239230

[7] Masroor S: Collateral damage of COVID-19 pandemic: delayed medical care. J Card Surg 2020; 35(6): 1345-7. 32419177 10.1111/jocs.14638 PMC7276840

[8] Collins CM, Davenport DL, Talley CL, Bernard AC: Appendicitis grade, operative duration, and hospital cost. J Am Coll Surg 2018; 226(4): 578-83. 29391281 10.1016/j.jamcollsurg.2017.12.046

[9] Goyal P, Choi JJ, Pinheiro LC, et al: Clinical characteristics of COVID-19 in New York City. N Engl J Med 2020; 382(24): 2372-4. 32302078 10.1056/NEJMc2010419 PMC7182018

[10] Saez M, Tobias A, Varga D, Barceló MA: Effectiveness of the measures to flatten the epidemic curve of COVID-19. The case of Spain. Sci Total Environ 2020; 20(727): 138761. 10.1016/j.scitotenv.2020.138761 PMC7166106 32330703

[11] Smith AC, Thomas E, Snoswell CL, et al: Telehealth for global emergencies: implications for coronavirus disease 2019 (COVID-19). J Telemed Telecare 2020; 26(5): 309-13. 32196391 10.1177/1357633X20916567 PMC7140977

[12] Romero J, Valencia S, Guerrero A: Acute appendicitis during coronavirus disease 2019 (COVID-19): changes in clinical presentation and CT findings. J Am Coll Radiol 2020 8; 17(8): 1011-3. doi: 10.1016/j.jacr.2020.06.002. 32610104 PMC7321660

[13] Velayos M, Muñoz-Serrano AJ, Estefanía-Fernández K, et al: Influence of the coronavirus 2 (SARS-Cov-2) pandemic on acute appendicitis. An Pediatr 2020 8; 93(2): 118-22. doi: 10.1016/j.anpede.2020.04.010. PMC7328590 32837965

[14] Snapiri O, Rosenberg Danziger C, Krause I, et al: Delayed diagnosis of paediatric appendicitis during the COVID-19 pandemic. Acta Paediatr Int J Paediatr 2020 8; 109(8): 1672-6. doi: 10.1111/apa.15376. PMC7283758 32460364

[15] Collard M, Lakkis Z, Loriau J, et al: Antibiotics alone as an alternative to appendectomy for uncomplicated acute appendicitis in adults: changes in treatment modalities related to the COVID-19 health crisis. J Visc Surg 2020; 157: S33. 10.1016/j.jviscsurg.2020.04.014 PMC7181971 32362368

[16] Kvasnovsky CL, Shi Y, Rich BS, et al: Limiting operations for acute appendicitis in children: lessons learned from the U.S. epicenter of the COVID-19 pandemic. J Pediatr Surg 2020. 10.1016/j.jpedsurg.2020.06.024 PMC7309720 32620267

[17] Suwanwongse K, Shabarek N: Successful conservative management of acute appendicitis in a coronavirus disease 2019 (COVID-19) patient. Cureus 2020; 12(4). 10.7759/cureus.7834 PMC7250521 32467809

[18] Truche P, Bowder A, Lalla AT, et al: Perspectives on perioperative management of children's surgical conditions during the COVID-19 pandemic in low-income and middle-income countries: a global survey. World Journal of Pediatric Surgery. 2020; 3(3): e000187. Published 2020 Sep 15. doi: 10.1136/wjps-2020-000187. PMC7493076 38607942

[19] Pautrat K, Chergui N: SARS-CoV-2 infection may result in appendicular syndrome: chest CT scan before appendectomy. J Visc Surg 2020; 157(3): S63. 10.1016/j.jviscsurg.2020.04.007 PMC7158827 32387057

[20] Ashcroft J, Hudson VE, Davies RJ: COVID-19 gastrointestinal symptoms mimicking surgical presentations. Ann Med Surg 2020 56: 108-9. doi: 10.1016/j.idcr.2020.e00860. PMC7311923 32632349

[21] Abdalhadi A, Alkhatib M, Mismar AY, Awouda W, Albarqouni L: Can COVID 19 present like appendicitis? IDCases 2020 6 2;21: e00860. doi: 10.1016/j.idcr.2020.e00860. PMC7265835 32523872

[22] Cai X, Ma Y, Li S, Chen Y, Rong Z, Li W: Clinical characteristics of 5 COVID-19 cases with non-respiratory symptoms as the first manifestation in children. Front Pediatr 2020 5 12;8: 258. doi: 10.3389/fped.2020.00258. PMC7235428 32574284

